# Supplementary material for: Detection of Filoviruses in Bats in Vietnam
Source: Viruses. 2023 Aug 23;15(9):1785. doi: 10.3390/v15091785 (PMC10534609; doi:10.3390/v15091785)
Supplement: Supplementary file 1 [file viruses-15-01785-s001.zip › Figure S1 Cutoff calculation for ELISA_revised.pdf]

Cutoff calculation for ELISA

In the absence of confirmed filovirus-negative bat serum, we used the following methods to determine the cutoff value. The first step was to normalize the raw data on optical density obtained from different plates/runs. To do this, we added a control sample to each plate, which was included in the manufacturer's kit and contained 1 unit/ml of human antibodies. Then, we divided the optical density data from each plate by the optical density of the control sample on that plate. The cutoff calculation was performed in R for each bat genus separately.

Then, we ordered the normalized optical density for each genus of bats in ascending order and performed a change point analysis. Change point analysis was carried out using the 'EnvCpt' package [33]. Change-point analysis is a statistical analysis that can detect a series of values, a step indicating a change. Such a change could be observed in a mixture of two datasets with different parameters. Since we assume that the mean absorbance values of seropositive samples will be significantly higher than the mean absorbance value of seronegative samples, change point analysis is useful for detecting this difference [34]. The change point splits the data into two sets. We extracted the dataset with the lowest optical density and approximated it with a parametric model. If the data were normally distributed, we used the 'fitdistrplus' package [35] to estimate the distribution parameters. If the data were skewed and approximated by a skew-normal distribution, then we used the 'sn' package [36] to estimate the distribution parameters. The 99.9% quantile of the distribution was considered the cutoff level, which was estimated using parametric bootstrapping with 10,000 iterations.

The cutoff calculation results for seropositive datasets are presented below:

Dataset 1

Normalized optical density for *Rousettus* sp., ELISA with Marburg virus glycoprotein antigen. The dataset includes 53 observations of *R. leschenaultii* and 8 observations of *R. amplexicaudatus*.

Change point analysis:

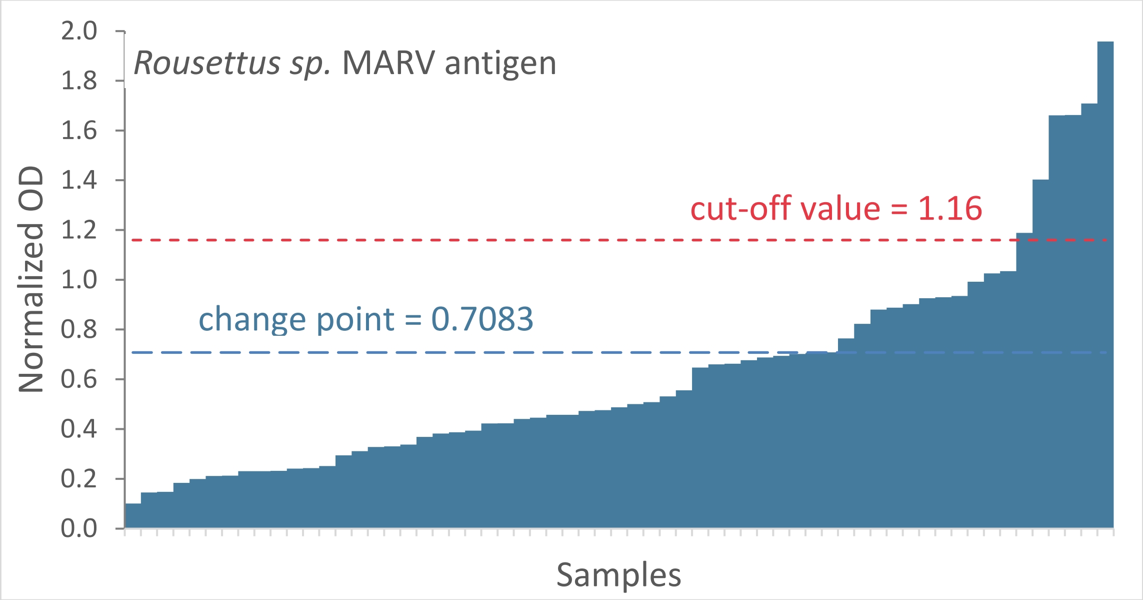

Cut-off calculation:

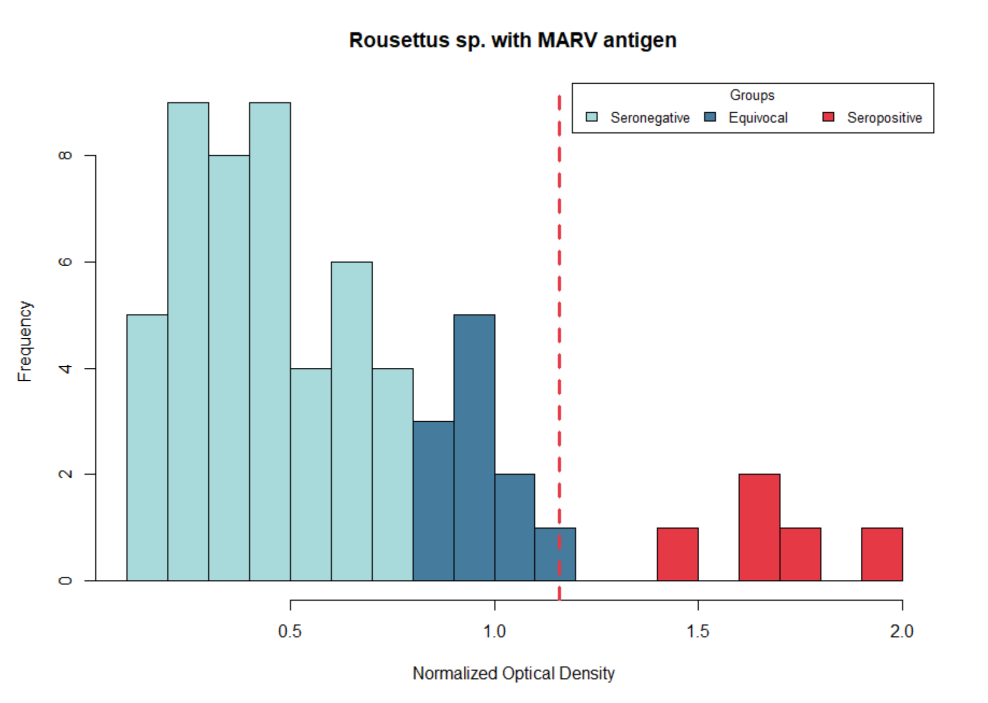

Seronegatives were approximated with skew-normal distribution with the following parameters*:

Mu	0.162
Sigma	0.305
Shape	5.772

Bootstraped 99.9% quantile of the distribution: 1.16

* *Mu* is the location parameter, *Sigma* is the scale parameter, *Shape* is the shape parameter of the Skew-normal distribution

DATASET 2

Normalized optical density for *Rousettus* sp., ELISA with Ebolaviruses glycoprotein antigen.
The dataset includes 53 observations of *R. leschenaultii* and 8 observations of *R. amplexicaudatus*.

Change point analysis:

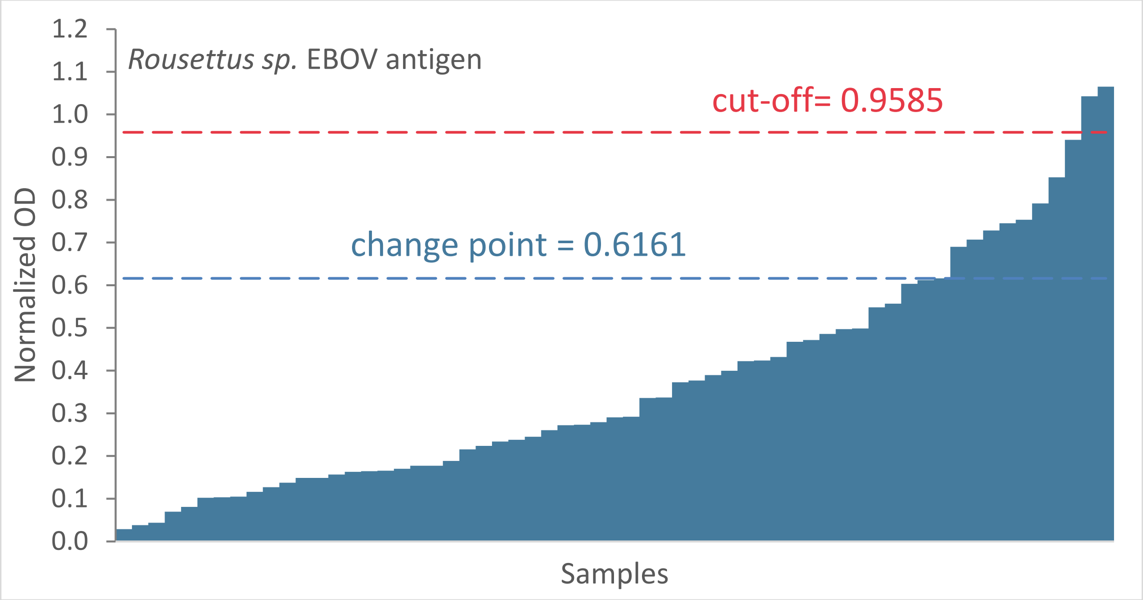

Cut-off calculation:

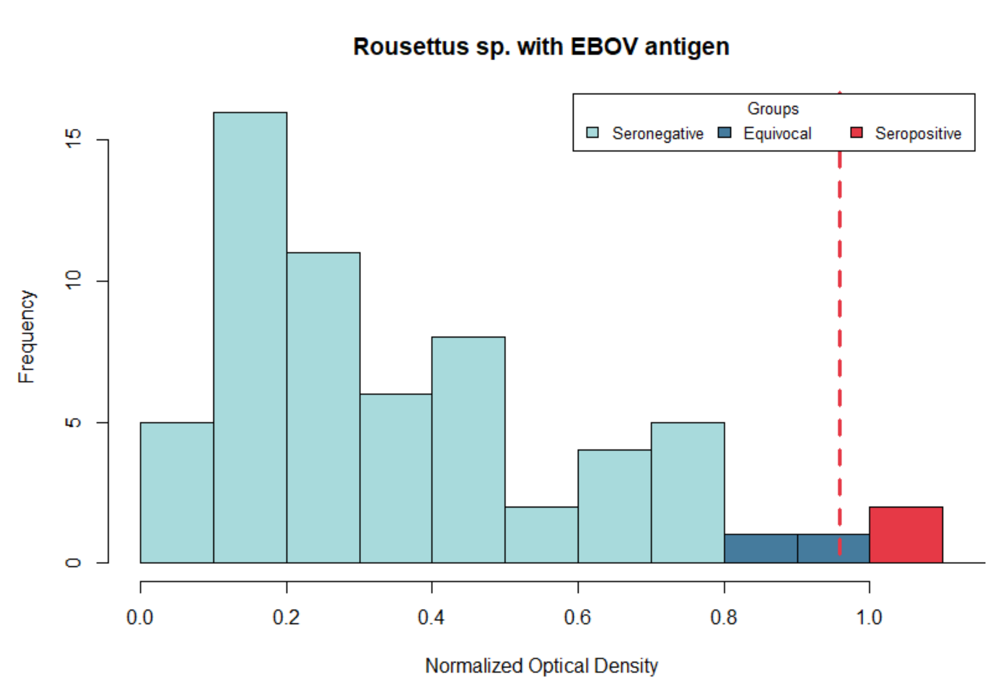

Seronegatives were approximated with skew-normal distribution with the following parameters*:

Mu	0.059
Sigma	0.275
Shape	6.965

Bootstraped 99.9% quantile of the distribution:

0.9585

* *Mu* is the location parameter, *Sigma* is the scale parameter, *Shape* is the shape parameter of the Skew-normal distribution

DATASET 3

Normalized optical density for *Eonycteris spelaea*, ELISA with Marburg virus glycoprotein antigen. The dataset includes 30 observations of *E. spelaea*

Change point analysis:

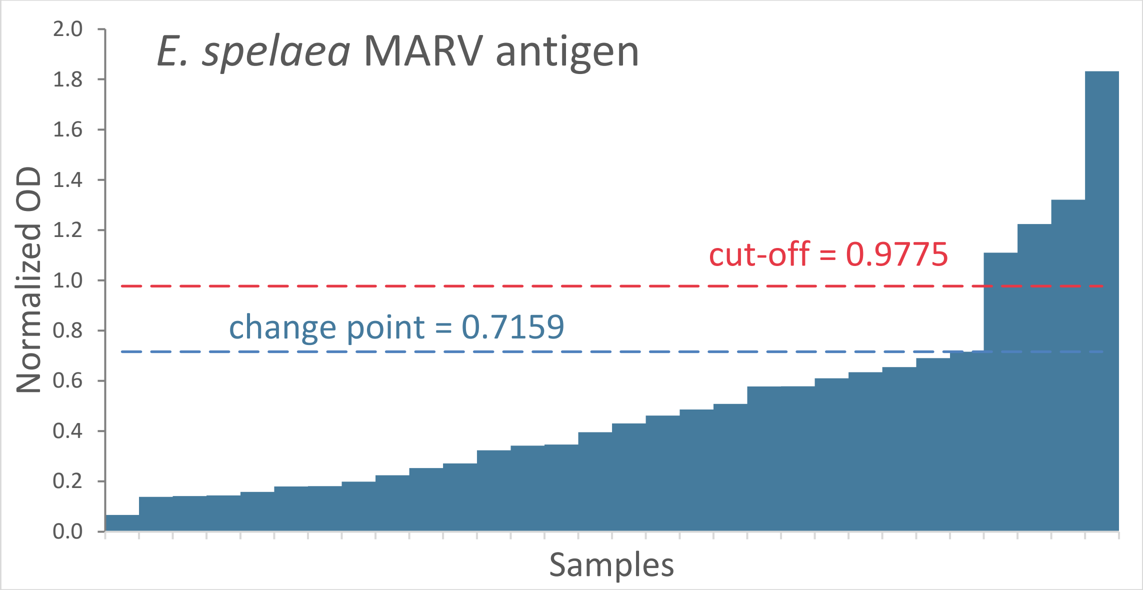

Cut-off calculation:

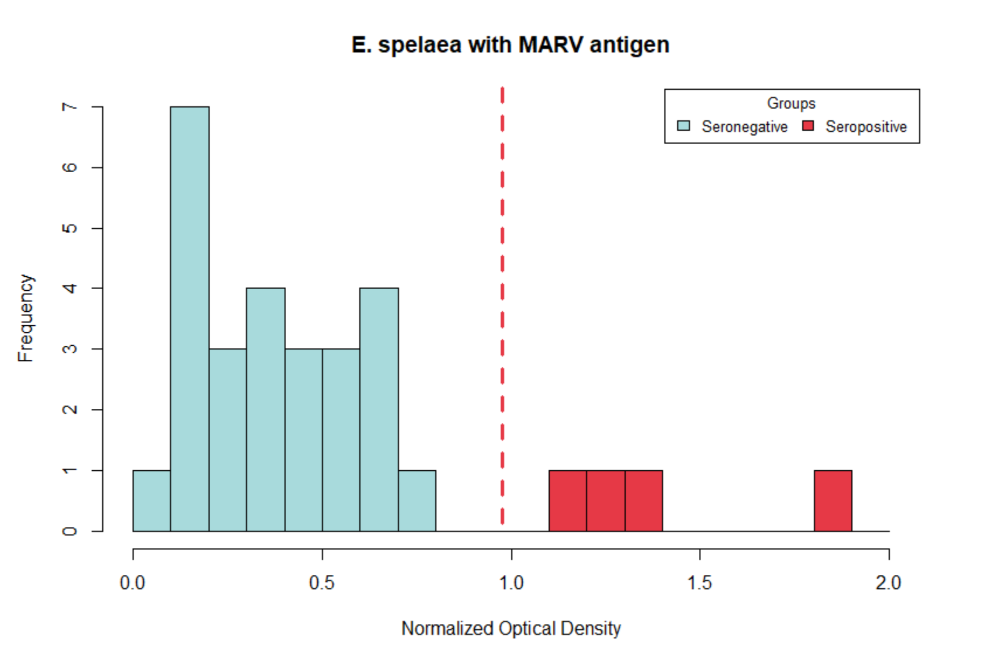

Seronegatives were approximated with normal distribution with the following parameters*:	
Mean	0.059
SD	0.275
Bootstraped 99.9% quantile of the distribution:	
0.9775	

DATASET 4

Normalized optical density for *Eonycteris spelaea*, ELISA with Ebolaviruses glycoprotein antigen. The dataset includes 30 observations of *E. spelaea*

Change point analysis:

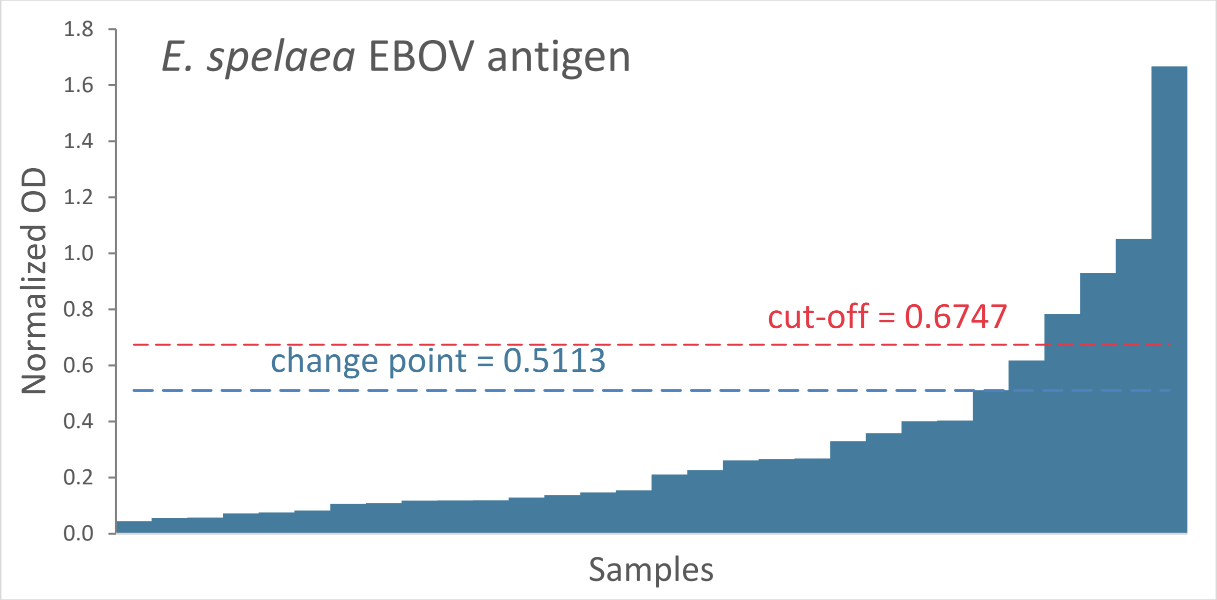

Cut-off calculation:

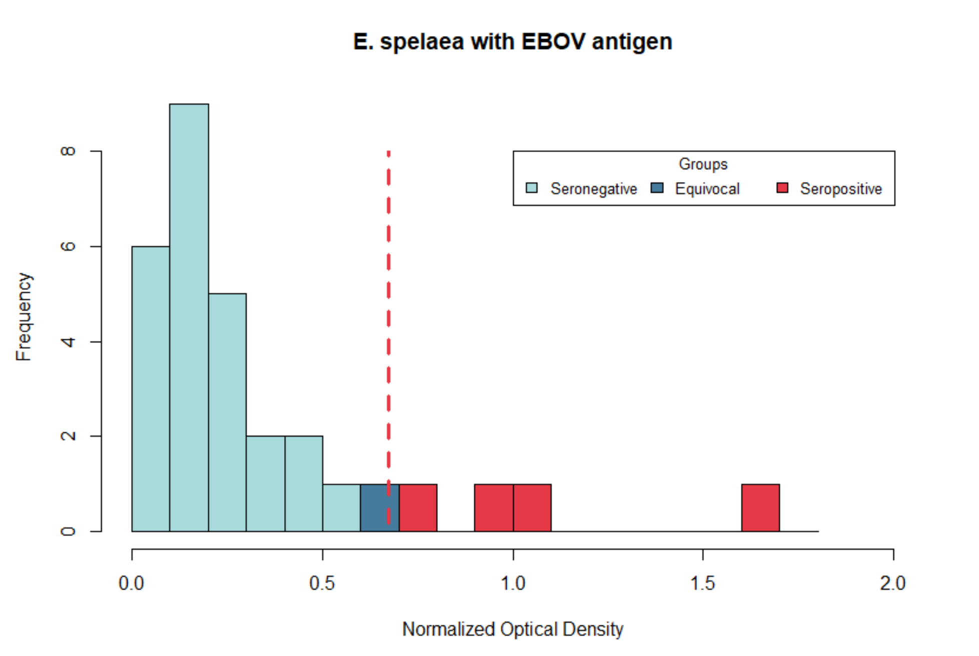

Seronegatives were approximated with skew-normal distribution with the following parameters*:	
Mu	0.043
Sigma	0.193
Shape	183.45
Bootstraped 99.9% quantile of the distribution:	
0.6747	

* *Mu* is the location parameter, *Sigma* is the scale parameter, *Shape* is the shape parameter of the Skew-normal distribution

DATASET 5

Normalized optical density for *Cynopterus sp.*, ELISA with Marburg virus glycoprotein antigen. The dataset includes 39 observations of *C. sphinx*, and 2 observations of *C. horsfieldi.*

Change point analysis:

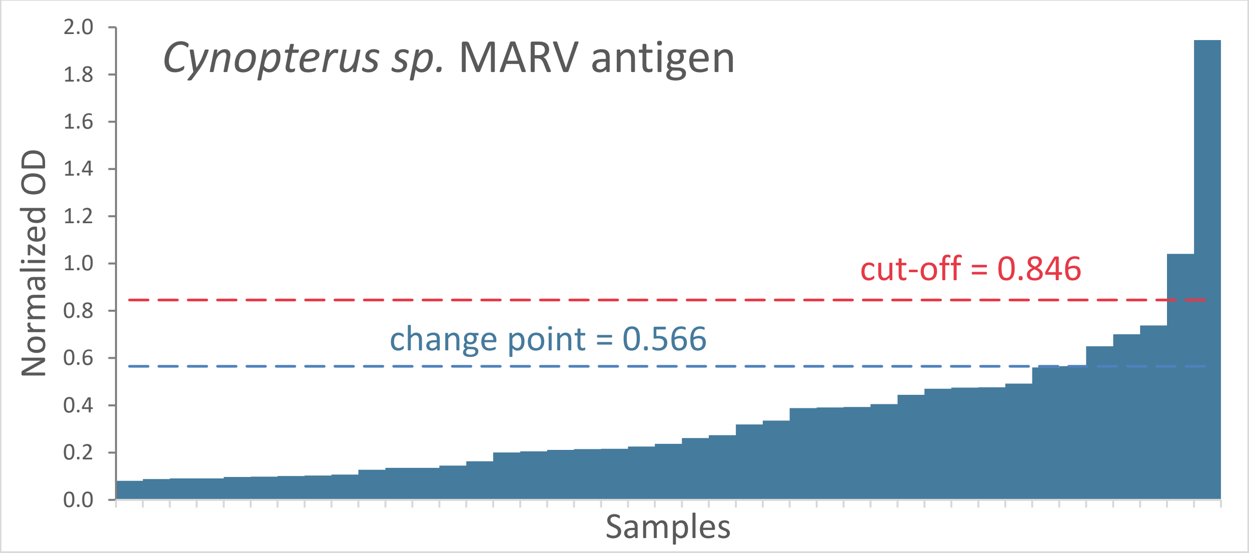

Cut-off calculation:

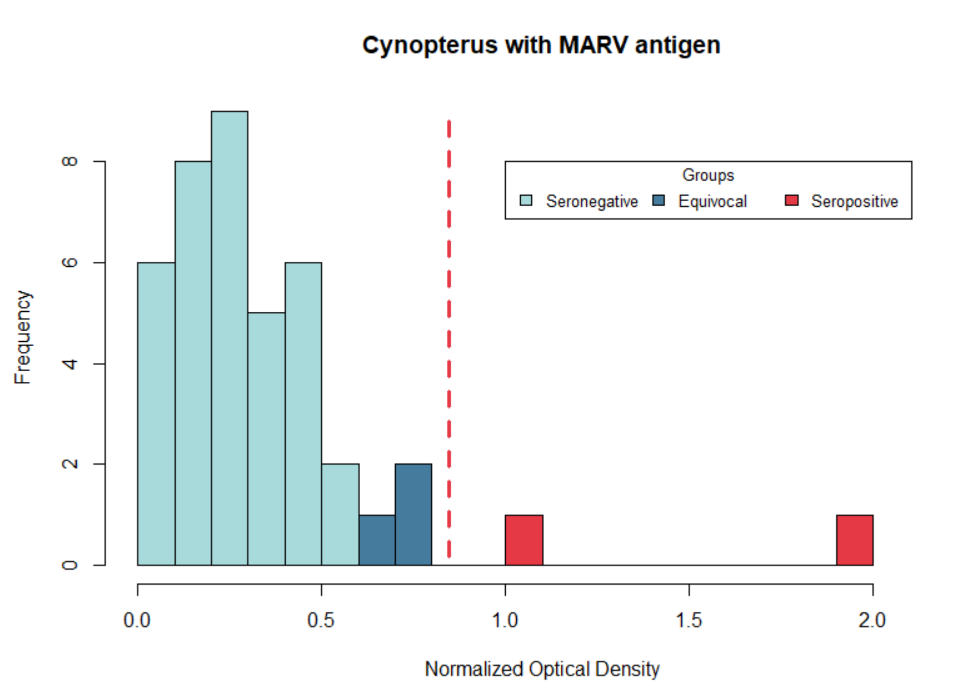

Seronegatives were approximated with skew-normal distribution with the following parameters*:

Mu	0.0784
Sigma	0.2347
Shape	183.446

Bootstraped 99.9% quantile of the distribution:

0.846

* *Mu* is the location parameter, *Sigma* is the scale parameter, *Shape* is the shape parameter of the Skew-normal distribution

DATASET 6

Normalized optical density for *Cynopterus sp.*, ELISA with Ebolaviruses glycoprotein antigen. The dataset includes 39 observations of *C. sphinx*, and 2 observations of *C. horsfieldi.*

Change point analysis:

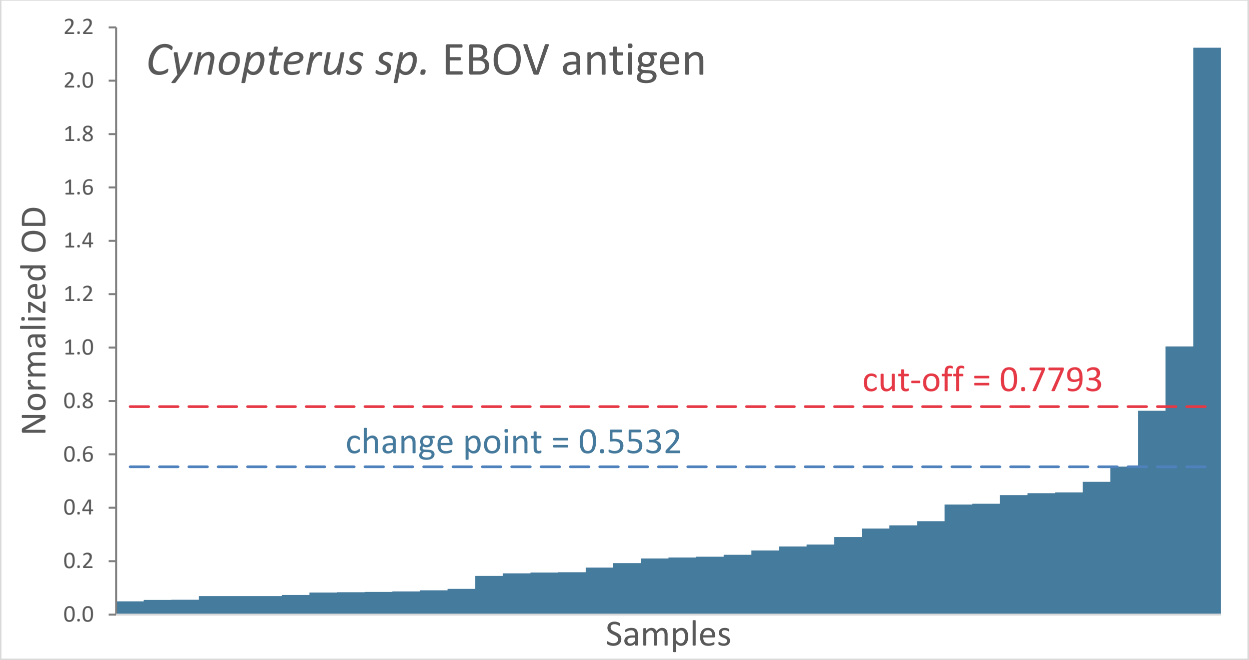

Cut-off calculation:

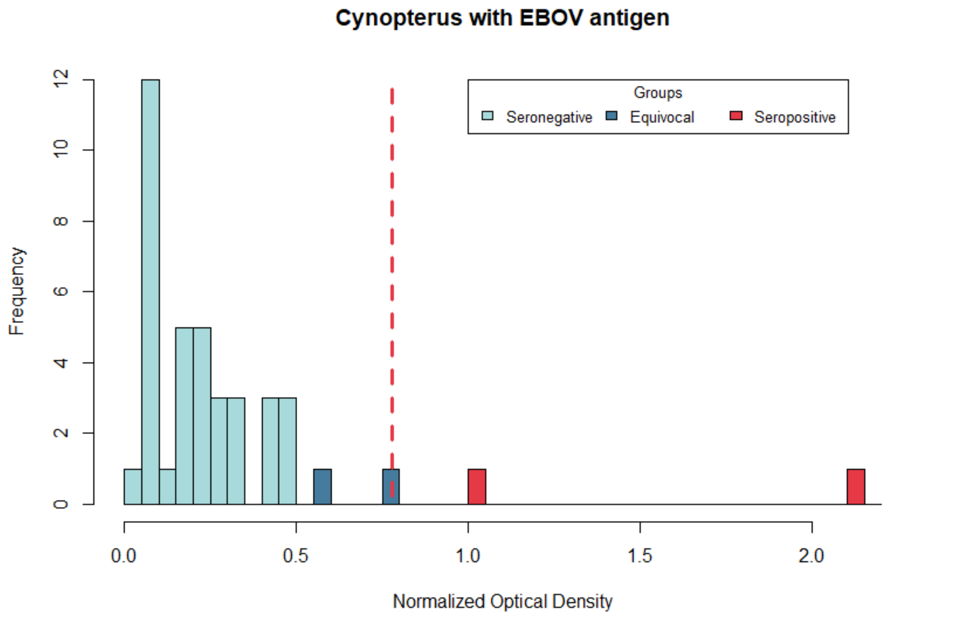

Seronegatives were approximated with skew-normal distribution with the following parameters*:	
Mu	0.0477
Sigma	0.2236
Shape	183.45
Bootstraped 99.9% quantile of the distribution:	
0.7793	

* *Mu* is the location parameter, *Sigma* is the scale parameter, *Shape* is the shape parameter of the Skew-normal distribution
