# Supplementary material for: Detection of Filoviruses in Bats in Vietnam
Source: Viruses. 2023 Aug 23;15(9):1785. doi: 10.3390/v15091785 (PMC10534609; doi:10.3390/v15091785)
Supplement: Supplementary file 1 [file viruses-15-01785-s001.zip › Table S1. Primer sequences and conditions for PCR assays.pdf]

Table S1. Primer sequences and conditions for PCR assays.

Primers	Stage	5’-3’ primer sequence	PCR conditions	
Nested PCR assay for screening of filoviruses , He B. et al [18]				
FV-F1<br>FV-R1	1st round of PCR	GCMTTYCCIAGYAAYATGATGG<br>GTDATRCAYTGRTTRTCHCCCAT	95°C for 5 min<br>95°C for 30 s<br>54°C for 30 s<br>72°C for 40 s<br>72°C for 5 min	35 cycles
FV-F2<br>FV-R2	2nd round of PCR (351 bp)	TDCAYCARGCITCDTGGCAYC<br>GIGCACGADATRWIGTCC	95°C for 5 min<br>95°C for 30 s<br>54°C for 30 s<br>72°C for 40 s<br>72°C for 5 min	30 cycles
Nested PCR for screening of filoviruses, Goldstein T. et al [15]				
Filo-Mod-FWD<br>Filo L. conR	1st round of PCR	TITTYTCHVTICAAAAICAYTGGG<br>ACCATCATRTTRCTIGGRAAKGCTTT	95°C for 5 min<br>95°C for 15 s<br>55°C for 60 s<br>72°C for 60 s<br>72°C for 5 min	40 cycles
Filo-Mod-FWD<br>Filo-Mod-RVS	2nd round of PCR (680 bp)	TITTYTCHVTICAAAAICAYTGGG<br>GCYTCISMIAIIGTTTGIACATT	95°C for 5 min<br>95°C for 15 s<br>58°C for 60 s<br>72°C for 60 s	3 cycles
			95°C for 15 s<br>55°C for 60 s<br>72°C for 60 s	3 cycles
			95°C for 15 s<br>53°C for 60 s<br>72°C for 60 s	3 cycles
			95°C for 15 s<br>50°C for 60 s<br>72°C for 60 s<br>72°C for 5 min	20 cycles
PCR for screening MARV and MARV-like viruses, Aiginin A. et al [25]				
Marburg F V2<br>Marburg R V2<br>(398 bp)		CVTATCGTGTCAGAAATGTCCA<br>ATTDGCACAATCAGGTGGGT	95°C for 5 min<br>95°C for 15 s<br>58°C for 60 s<br>72°C for 60 s	3 cycles
			95°C for 15 s<br>55°C for 60 s<br>72°C for 60 s	3 cycles
			95°C for 15 s<br>53°C for 60 s<br>72°C for 60 s	3 cycles
			95°C for 15 s<br>50°C for 60 s<br>72°C for 60 s<br>72°C for 5 min	33 cycles
